# The Altered Neonatal CD8^+^ T Cell Immunodominance Hierarchy during Influenza Virus Infection Impacts Peptide Vaccination

**DOI:** 10.3390/v16081271

**Published:** 2024-08-09

**Authors:** Luke Heil, Samantha Jewell, J. Louise Lines, Beth A. Garvy

**Affiliations:** 1 Department of Microbiology, Immunology, and Molecular Genetics, University of Kentucky, Lexington, KY 40536, USA; luke.heil@uky.edu (L.H.); samantha.jewell@nsc.edu (S.J.); janet.l.lines@dartmouth.edu (J.L.L.); 2 Department of Physical and Life Sciences, Nevada State University, Henderson, NV 89002, USA; 3 Department of Microbiology and Immunology, Geisel School of Medicine at Dartmouth, Hanover, NH 03755, USA; 4 Division of Infectious Diseases, University of Kentucky, Lexington, KY 40536, USA

**Keywords:** CD8 T cells 1, neonate 2, influenza virus 3

## Abstract

Neonates are more susceptible to influenza virus infection than adults, resulting in increased morbidity and mortality and delayed clearance of the virus. Generating effective CD8^+^ T cell responses may be important for improving vaccination outcomes in vulnerable populations, but neonatal T cells frequently respond differently than adult cells. We sought to understand CD8^+^ T cell specificity and immunodominance during neonatal influenza infection and how any differences from the adult hierarchy might impact peptide vaccine effectiveness. Neonatal C57BL/6 mice displayed an altered CD8^+^ T cell immunodominance hierarchy during influenza infection, preferentially responding to an epitope in the influenza protein PA rather than the co-dominant adult response to NP and PA. Heterosubtypic infections in mice first infected as pups also displayed altered immunodominance and reduced protection compared to mice first infected as adults. Adoptive transfer of influenza-infected bone-marrow-derived dendritic cells promoted an NP-specific CD8^+^ T cell response in influenza-virus-infected pups and increased viral clearance. Finally, pups responded to PA (224–233), but not NP (366–374) during peptide vaccination. PA (224–233)-vaccinated mice were not protected during viral challenge. Epitope usage should be considered when designing vaccines that target T cells when the intended patient population includes infants and adults.

## 1. Introduction

Neonates and children are vulnerable to influenza virus infection, suffering significant morbidity and even mortality as a result of this common infection [1,2,3]. This remains the case despite yearly vaccination efforts [4,5]. Children under 6 months of age cannot be vaccinated for influenza, and even after that period, two injections are required to confer protection instead of the one injection adults require [6]. As a result, influenza causes more hospitalizations and deaths in American children than any other vaccine-preventable disease [2].

The neonatal immune response exhibits a variety of differences from the adult response that may underlie the observed vulnerability of infants to influenza virus infection [7,8,9,10]. Using mouse models, we have shown that CD4^+^ and CD8^+^ T cell migration into the lungs is delayed in mouse pups during influenza virus infection [11]. We have previously shown that during *Pneumocysitis murina* infection, lower expression of ICAM-1 and VCAM-1 on neonatal endothelial cells is likely related to this defect in migration [12]. Additionally, neonatal T cells accumulate in the interstitium of the lungs instead of moving into the alveolar spaces as is seen in adults [11]. This is similar to the interstitial pneumonia observed in severe human infant influenza virus infections [13,14]. These diminished T cell populations correlate with changes in function, with neonatal T cells displaying reduced cytotoxicity and cytokine production compared to adult cells in a variety of viral illnesses [8,9,15]. Intriguingly, neonatal T cells can respond robustly like adult T cells given increased costimulation [16,17]. We have seen in our murine model of influenza infection that with this diminished immune function, pups struggle to clear even minute quantities of virus [11]. Understanding the functional aspects of the neonatal influenza virus response is necessary for effectively designing specific treatments that improve infection outcomes while avoiding excess inflammation in this fragile population [18].

Significant effort is being devoted to increasing the protection of influenza virus vaccination by generating responses that are cross-reactive against more than one virus strain [19,20,21]. New avenues of research focus on vaccines that stimulate B or T cell responses that target conserved regions of the virus, such as the hemagglutinin (HA) stalk, an influenza virus surface protein used for viral entry, or the nucleoprotein (NP), an RNA-binding protein critical for viral replication [22,23]. Conserved regions of viral proteins typically cannot mutate readily without losing vital functions, so it is difficult for viruses to escape immune responses to these epitopes [24]. 

CD8^+^ T cells are one of the targets of these vaccination efforts, but CD8 T cell responses are often considered in conjunction with B cell responses rather than as a standalone vaccine response [25,26]. In humans, CD8^+^ memory confers cross-strain protection against severe influenza morbidity and mortality, but not the complete neutralizing protection afforded by antibody mediated monovalent vaccines [27]. CD8^+^ T cells, however, often target epitopes that cannot readily mutate without compromising viral function [26]. This leaves CD8^+^ vaccination as a potentially important means of bypassing antigenic drift while reducing disease severity and conferring longer-lasting protection. 

During T cell receptor (TCR) generation, a diverse repertoire of T cell specificities is created [28]. During an immune response, however, most of the expanded T cell population derives from clones specific for a few of the many potential epitopes in a given antigen, a principle known as immunodominance [29,30,31]. In C57BL/6 mice, the peptides NP (366–374) and PA (224–233) are known to be co-dominant in adult A/PR/8/34 (PR8) influenza virus infections, but it has been suggested that immunodominance can be shifted in neonates [9,32]. In respiratory syncytial virus (RSV) infections, neonates respond co-dominantly to epitopes from the M2 and M proteins compared to the strong M2-restricted response in adult mice [33]. Similarly, in influenza virus infection, there is evidence that pups do not respond to the adult dominant epitope, NP (366–374) [32]. If vaccines targeting CD8^+^ T cells are added to conventional influenza virus vaccine strategies, it is important to ascertain whether these vaccines will function the same in neonates as in adults. As such, we were interested in confirming whether neonatal mice could respond to NP (366–374) similarly to adult mice and investigating other peptides to establish a likely immunodominant peptide for neonatal CD8^+^ T cell responses. We also examined whether peptide vaccination could offer protection against influenza virus challenge in neonates.

Using an acute model of influenza virus infection, this study identifies what may be the immunodominant peptide in neonatal C57BL/6 mice. Responses were compared between adult mice and pups to evaluate CD8^+^ specificity in primary and secondary responses. We found the primary neonatal response to be strongly biased towards polymerase acidic protein (PA) (224–233), rather than the adult co-dominant response to NP (366–374) and PA (224–233). CD8^+^ T cell specificity is less predictable in the secondary infections of mice first infected as neonates compared to mice first infected as adults. Some mirror the strong dominance of NP (366–374)-specific CD8^+^ T cells, while others exhibit NP and PA co-dominance or even PA dominance alone. Regardless of hierarchy, CD8^+^ memory was less effective in mice first infected as pups. Additionally, we found that transfer of PR8-loaded bone-marrow-derived dendritic cells (BMDCs) improved viral clearance in pups as well as promoting NP (366–374)-specific CD8^+^ T cells. Finally, although we were able to generate a PA (224–233)-specific response to a peptide-loaded BMDC vaccine, this response was not protective during influenza virus challenge. We believe these data support previous findings about neonatal T cell specificity [32,33] and demonstrate that the immunodominance hierarchy can be manipulated. These data also show the importance of examining the neonatal period separately from adult responses when examining an immunodominance hierarchy and developing subunit vaccines. 

## 2. Materials and Methods

### 2.1. Mice, Viral Stocks, and Influenza Virus Peptides

C57BL/6J mice were purchased from Jackson Labs (Bar Harbor, ME, USA). Timed pregnancies were generated by co-housing female mice for two weeks to synchronize estrus. All mice were maintained at the University of Kentucky Division of Laboratory Animal Research facilities with the approval of the University of Kentucky Institutional Animal Care and Use Committees (IACUC) and Institutional Biosafety Committees.

Influenza viruses, A/Puerto Rico/8/34 (H1N1) (PR8) and A/X-31(H3N2) (HKx31) were grown in 10-day-old specific pathogen-free embryonated chicken eggs (Charles River, North Franklin, CT, USA) as has been described before [34]. Both viral stocks were verified to contain only influenza virus by the University of Missouri Research and Diagnostic Laboratory (RADIL). Viral titers were measured in egg infectious doses (EID_50_) as described previously [11].

Custom peptides were purchased from GenScript (Piscataway, NJ, USA). Peptide sequences from PR8 recognized in H2(b) mice were NP (366–374) ASNENMETM, PA (224–233) SSLENFRAYV, PB1 (703–711) SSYRRPVGI, and PB1-F2 (62–70) LSLRNPILV [30,35,36,37].

### 2.2. Infections

Mice were given LD_10_ doses of PR8 or X-31 intranasally under isoflurane anesthesia. For PR8, this was equivalent to 2.5 egg infection dose (EID)_50_/g of body weight for adults and 0.25 EID_50_/g of body weight in pups. Both male and female mice were used. For HKx31, this was equivalent to 10 EID_50_/g of body weight in adults and 1 EID_50_/g of body weight in pups. Following infections, all mice were monitored daily for body weight and euthanized in accordance with the University of Kentucky IACUC’s criteria of body weight loss or failure to thrive. 

For secondary infections, lethal dose was delivered intranasally under isoflurane anesthesia. This corresponded to 25 (EID)_50_/g of body weight for PR8 and 100 (EID)_50_/g of body weight for HKx31. Following infections, all mice were monitored daily for body weight and euthanized in accordance with the University of Kentucky IACUC’s criteria of body weight loss and body condition score.

### 2.3. Cell Isolation

Lungs were lavaged five times with cold HBSS/3 mM EDTA to extract alveolar cells. Lungs were excised and minced before digestion at 37 °C with 50 U/mL DNAse (Sigma Aldrich, St. Louis, MO, USA) and 1 mg/mL collagenase (Sigma Aldrich) in RPMI with 3% FCS. Single cell suspensions of lungs and tracheobronchial lymph nodes (TBLN) were generated by pressing tissues though 70 μm cell strainers (BD Biosciences, San Jose, CA, USA). Red blood cells were lysed using ammonium chloride/potassium (ACK) lysis buffer. Cells were then washed and resuspended in HBSS. CD8^+^ T cells were isolated from lymph nodes or lungs using negative selection CD8 T cell mini-columns (R&D Systems, Minneapolis, MN, USA) according to manufacturer’s instructions. For some experiments, CD8^+^ T cells were isolated by positive selection using CD8^+^ EasySep microbeads (StemCell Technologies, Cambridge, MA, USA) according to manufacturer’s instructions.

### 2.4. Flow Cytometry

Surface staining was performed on 5 × 10^5^ to 10^6^ cells with fluorochrome conjugated antibodies or MHC-I tetramers (NIH Tetramer Core Facility at Emory University) in phosphate-buffered saline containing 0.1% BSA and 0.02% NaN3 (PBA) for 30 min at room temperature. Cells were fixed with 5% neutral buffered formalin (Sigma Aldrich) at room temperature for 20 min before being washed with HBSS. 

For intracellular staining, cells were stimulated for 4 h at 37 °C with 50 ng/mL PMA (Enzo Life Sciences, Farmingdale, NY, USA) and 1 μg/mL of ionomycin (Sigma Aldrich). Brefeldin A (Sigma Aldrich) (10 μg/mL) was added after 2 h to trap cytokines in the cytoplasm. Cells were surface stained as described above with the addition of 10 μg/mL Brefeldin A to the PBA. Cells were fixed with 5% neutral buffered formalin (Sigma Aldrich), permeabilized with saponin (5 mg/mL), and stained for 30 min with antibodies specific for IFNγ. Analysis was performed by flow cytometry with an LSRII cytofluorometer (BD Biosciences, Mountain view, CA). A total of 50,000 events were collected for each sample and analysis was performed with FlowJo software (Tree Star, Inc., Ashland, OR, USA).

Antibodies used in this study were fluorochrome-conjugated anti-mouse CD4, clone GK1.5; anti-mouse CD8, clone 53-6.7; anti-mouse CD44, clone IM7; anti-mouse CD62L, clone MEL-14; and anti-mouse IFNγ, clone XMG1.2 (all from Thermo Fisher Scientific, Waltham, MA, USA). 

### 2.5. Antigen Presentation Assay

Bone-marrow-derived dendritic cells (BMDCs) were generated by flushing the femurs of adult, female, C57BL/6 mice with cold RPMI (ThermoFisher) to isolate bone marrow. Red blood cells were lysed with ACK as described above and bone marrow was plated with 10 mL RPMI containing 10% FCS, 0.1% 2-Mercaptoethanol (Sigma), and 1 ng/mL GM-CSF (Peprotech, Rocky Hill, NJ, USA). After 10 days, BMDCs were washed and incubated for 1 h with PR8 at a 0.1 multiplicity of infection, peptides, or left uninfected. Treated BMDCs were cultured 1:5 with CD8^+^ T cells isolated from lymph nodes of mice 10 days post-infection. After 3 days, supernatants were analyzed for IFNγ by sandwich ELISA per the manufacturer’s instructions (ThermoFisher). Optical density of plates was read at 405 nm using a Bio-Tek Instruments, Inc. (Winooski, VT, USA) plate reader and KC Junior software version 1.22. 

### 2.6. Adoptive Transfer of PR8-Infected or Peptide-Pulsed BMDCs

BMDCs were generated as described above. After 9 days, BMDCs were stimulated with 50 ng/mL LPS for 1 day. Then, influenza-virus-infected or control BMDCs were washed and incubated with 10^7^ EID PR8 or HBSS for one hour at 37 °C followed by washing and incubation for an additional 3 h at 37 °C. BMDCs were washed before adoptive transfer. Peptide-pulsed, LPS-stimulated DCs were incubated for 1 h with 1 mM peptide and then washed before adoptive transfer. In total, 10^4^ BMDCs were transferred intraperitoneally (i.p.) to pups on day 0 of infection or vaccination. A total of 5 × 10^4^ BMDCs were transferred to adults on day 0 of vaccination.

### 2.7. Determination of Viral Titers

Lungs were frozen at −80 °C until analyzed for viral burden. Viral titers were determined by plaque assay on Madin Darby Canine Kidney (MDCK) cells (ATCC, Manassas, VA, USA). Cells were grown to confluence in six-well plates in Dulbecco’s modified Eagle’s medium (DMEM) (ATCC) supplemented with non-essential amino acids (Thermo Fisher Scientific) and 10% heat-inactivated FCS (Atlas Biologicals, Fort Collins, CO, USA). Ten-fold dilutions of lung homogenate were incubated with the cells for 1 h at 37 °C. The cells were then washed and overlaid with serum-free DMEM in 1% Bacto Agar with 1% trypsin (Sigma-Aldrich). Three days later, the cells were fixed with 20% acetic acid and the overlay removed. Plaques were counted after staining with crystal violet. The limit of detection for this assay was 10 plaque-forming units (PFU)/lung.

### 2.8. Statistics

Data represent the mean ± SD or SEM for 3–10 mice per group and at least 2 separate experiments. Statistics calculations were performed with SigmaPlot software version 15.0 (San Jose, CA, USA). Student’s *t*-tests or 2-way analysis of variance (ANOVA) was performed to test for differences followed by Holm–Sidak post hoc test for pairwise comparisons. Mann–Whitney rank sum tests or Kruskal–Wallis one-way ANOVA on ranks were performed at each time point followed by Dunn’s pairwise post hoc test if normality or variance tests failed. *p* < 0.05 was considered statistically significant.

## 3. Results

### 3.1. Neonates Do Not Produce CD8^+^ T Cells Specific for NP (366–374) during Primary Influenza Virus Infection

**Figure 1:**
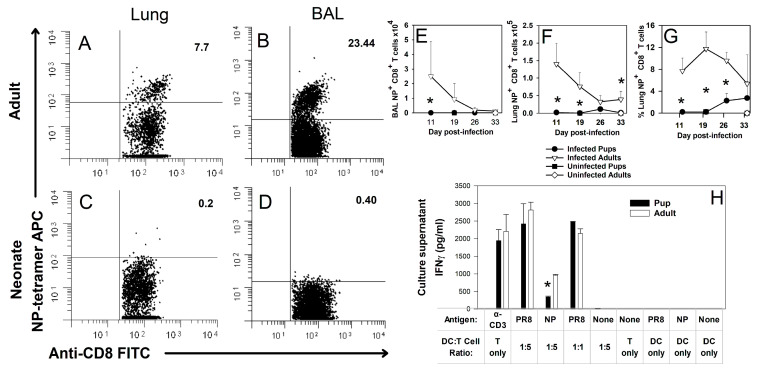
Pups do not respond to the adult dominant epitope NP (366–374). Mice at 8 weeks or 2 days of age were infected i.n. with an LD_10_ dose of PR8 strain of the influenza virus based on body weight. At day 10 of infection, BAL cells and lung digest cells were stained with an anti-CD8 antibody as well as H-2D(b) NP (366–374) tetramer and examined by flow cytometry. Representative scatter plots are shown for adult lungs (**A**) and the BAL (**B**) as well as pup lungs (**C**) and the BAL (**D**). The total numbers of tetramer^+^ and CD8^+^ cells are shown for the BAL (**E**) and lungs (**F**) of infected and uninfected pups and adults through day 34 after infection. The percentage of CD8^+^ cells that bound tetramer is also shown (**G**). CD8^+^ T cells were purified from adult or pup lymph nodes at 10 days post-infection and stimulated with BMDCs infected with PR8 or loaded with an NP peptide. Cells were cultured for 5 days, at which point supernatants were collected and analyzed for IFNγ by ELISA (**H**). The data represent the mean ± SD for two separate experiments and at least 10 mice per group. * *p* < 0.05 compared to adults at same time point.

The CD8^+^ immunodominance hierarchy for adult C57BL/6 mice has been clearly demonstrated during Influenza A PR/8/34 (PR8) infection [30,36], but less is known about neonatal CD8^+^ T cell specificity [32]. We wanted to confirm whether the dominant adult response to NP (366–374) could be recapitulated in neonatal mice responding to influenza virus challenge. We infected 2-day-old or 8-week-old mice with an LD_10_ dose of the PR8 influenza virus and analyzed whole lung digest and bronchoalveolar lavage (BAL) cells for antigen specificity by flow cytometry. At 10 days post-infection, pups displayed very few NP (366–374)-specific T cells as compared to adults in both the lung digest (Figure 1A,C) and BAL (Figure 1B,D). This is consistent with previous findings suggesting that pups do not respond to NP (366–374) during PR8 infection [32]. Our lab previously showed that adults clear an LD_10_ dose of the influenza virus by day 10 of infection while pups typically clear the influenza virus by day 14 of infection [11]. Following influenza virus infection, adult mice showed decreasing numbers of NP-specific CD8^+^ T cells in the BAL and lungs over time, as expected during the resolution phase of an infection (Figure 1E,F). Adult mice had a significantly higher proportion of NP-specific CD8^+^ T cells in the lungs at day 10, 20, and 26 than pups (Figure 1G). In the pups, small numbers of NP (366–374)-specific CD8^+^ T cells were detected 20–35 days post-infection in the lungs, although this population never approached the magnitude of the adult response during the period observed (Figure 1E–G). 

A functional assay was also used to verify the observed shift in the CD8^+^ immunodominance hierarchy. Two-day-old or eight-week-old mice were infected with an LD_10_ dose of the PR8 influenza virus before isolating CD8^+^ T cells by magnetic-activated cell sorting (MACS) from the lung digests of infected mice at day 10 of infection. CD8^+^ T cells and BMDCs were cultured together in the presence of the PR8 influenza virus or NP (366–374) peptide and IFNγ production was measured in the supernatant at day 3 of culture by ELISA. CD8^+^ T cells from pups responded to PR8 with similar levels of IFNγ as adults, but they displayed a significantly diminished response to NP (366–374) (Figure 1H). These data suggest that neonatal CD8^+^ T cells can respond to the influenza virus but that they do not robustly respond to NP (366–374).

### 3.2. Neonates Respond to PA (224–233) during Influenza Virus Infection

**Figure 2:**
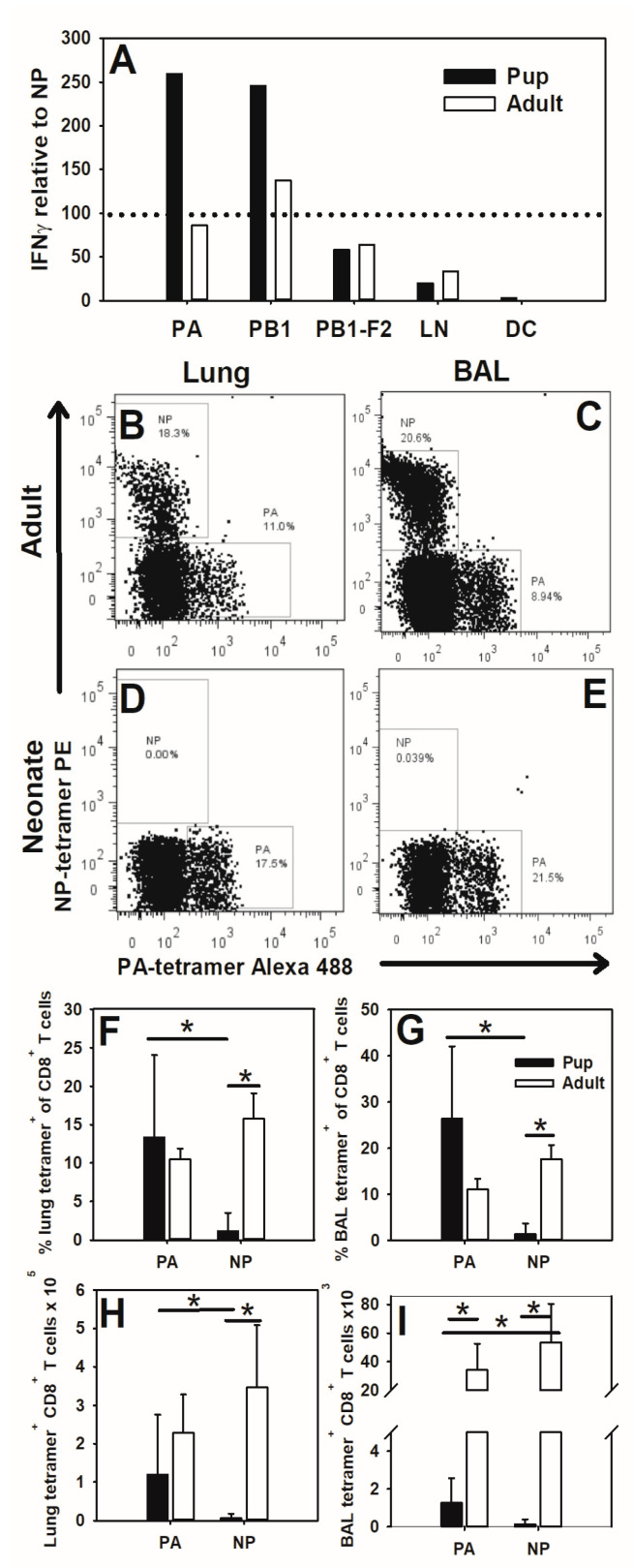
Pups respond to PA (224–233) and subdominant peptides. Adult or 2-day-old mice were infected i.n. with an LD_10_ dose of the PR8 strain of the influenza virus. At day 10 post-infection, TBLN were obtained from infected mice and cells were co-cultured with peptides and BMDCs. At day 3, supernatants were taken and IFNγ concentration was measured by ELISA. IFNγ production in response to PA, PB1, PB1-F2, and controls is shown as the mean of the ratio of each peptide response to the NP response of the same mouse for 10 pups and 4 adults. The dotted line represents 100% NP response (**A**). Cells from the whole lung digest as well as the BAL were also obtained at day 10 of infection and stained with an anti-CD8 antibody as well as H-2D(b) NP (366–374) and H-2D(b) PA (224–233) tetramers. Representative scatter plots are shown for CD8^+^ T cells from the adult lung digest (**B**) and BAL (**C**) as well as the pup lung digest (**D**) and BAL (**E**). Data are summarized as the percent of CD8^+^ T cells that are also tetramer-positive in the lung digest (**F**) and BAL (**G**). The total numbers of tetramer-positive CD8^+^ cells are shown for the lung digest (**H**) and BAL (**I**). The data show the mean ± SD for two separate experiments with at least eight mice per group. * *p* < 0.05.

Neonates are able to develop lung CD8^+^ T cell responses when infected with the influenza virus even though they lack appreciable NP (366–374)-specific T cells. This indicates that neonatal CD8^+^ T cells may be responding to other viral peptides. To test this, 2-day-old or 8-week-old mice were infected with an LD_10_ dose of the PR8 influenza virus. CD8^+^ T cells were isolated from the lymph nodes of pups and adults at day 10 of infection and stimulated with BMDCs loaded with NP (366–374), PA (224–233), PB1 subunit of the viral polymerase, PB1 (703–711), or PB1-F2 protein encoded by the +1 alternative open reading frame in the PB1 gene, PB1-F2 (62–70). IFNγ production was measured in supernatants at day 3 of culture by ELISA. IFNγ production in response to PA, PB1, PB1-F2, and controls was normalized for each mouse to the NP-stimulated response in the same mouse and averaged per group shown. Pup-lung-draining TBLN cells responded more robustly to PA (224–233) and PB1 (10–34) than to NP (366–374). Conversely, the adults displayed similar responses to NP, PA, and PB1 (Figure 2A).

To further explore this shift in immunodominance in vivo, 2-day-old or 8-week-old mice were infected with an LD_10_ dose of PR8 influenza and at day 10 post-infection, lung digest and BAL CD8^+^ T cells were analyzed for antigen specificity by flow cytometry using tetramers for NP (366–374) and PA (224–233). As seen in Figure 1, adults displayed robust NP-specific CD8^+^ T cell responses in the lung digest and BAL fluid (Figure 2B,C). PA (224–233)-specific CD8^+^ T cell populations were seen in adult lungs as well. Conversely, pups lacked NP (366–374)-specific CD8^+^ T cells, but populations of PA (224–233)-specific cells in both the lungs and alveolar spaces were clearly present (Figure 2D,E). Pups and adults have similar proportions of PA-specific CD8^+^ T cells in the lungs (Figure 2F) and BAL (Figure 2G), as well as similar total numbers of PA-specific cells in the lung (Figure 2H). As expected, neonates display fewer overall CD8^+^ T cells in the alveolar spaces at day 10 of influenza virus infection, which translates to lower overall numbers of antigen-specific CD8^+^ T cells in this location as well (Figure 2I). This is consistent with our previous work, suggesting that neonatal CD8^+^ T cells are unable to efficiently enter the airways during infection [11]. However, of the CD8^+^ T cells in the lung and BAL fluid at day 7 post-infection, 40% (SD = 8.3) and 72% (SD = 15.4%), respectively, were PA-specific in the pups. These data indicate that neonatal CD8^+^ T cells display a dominant PA (224–233) response instead of the adult co-dominant NP (366–374)- and PA (224–233)-specific response.

### 3.3. Neonatal Influenza Virus Infection Generates Weak T Cell Memory and Results in Altered CD8^+^ Specificity during Secondary Infection

**Figure 3:**
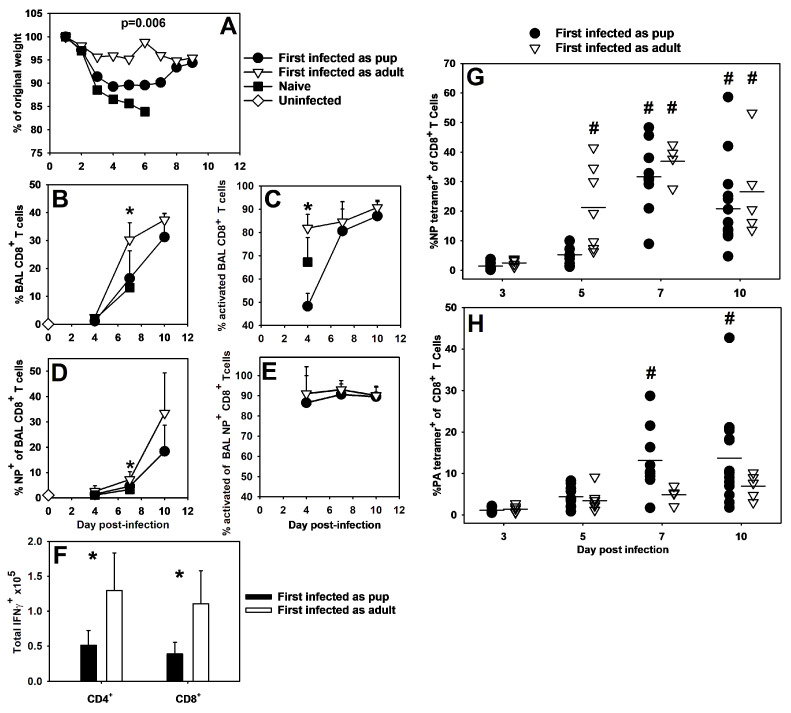
Mice infected as pups lose more weight and display altered secondary responses compared to mice infected as adults. Adult or 2-day-old C57BL/6 mice were infected i.n. with an LD_10_ dose of the X-31 strain of the influenza virus before being infected again with a lethal dose of the PR8 strain of the influenza virus 8 weeks later. The average percentage of starting body weight is shown for the secondary infection (**A**). The proportion of bronchoalveolar lavage (BAL) cells that were CD8^+^ T cells (**B**), proportion of CD8^+^ T cells in the BAL that were activated (CD44^+^ CD62L^lo^) (**C**), proportion of BAL CD8^+^ cells that were NP (366–374) tetramer^+^ (**D**), and percentage of tetramer^+^ CD8^+^ T cells in the BAL that were activated (CD44^+^ CD62L^lo^) (**E**) were assessed by flow cytometry. Total numbers of CD4^+^ and CD8^+^ lymphocytes that were also IFNγ^+^ are shown for the BAL at day 7 post-infection (**F**). The proportion of CD8^+^ cells that were NP (366–374) tetramer^+^ (**G**) or PA (224–233) tetramer^+^ (**H**) in the lung digest was assessed by flow cytometry. The data show the mean ± SD for two separate experiments with at least five mice per group. * *p* < 0.05 compared to mice first infected as pups at the same time point. # *p* < 0.05 compared to mice in the same group at day 3 of infection.

A significantly altered immunodominance hierarchy during a primary infection likely impacts the specificity of the memory response generated by that infection [30,38]. To explore this idea, our lab used heterosubtypic influenza virus infections to examine memory responses in mice first infected at 8 weeks of age or 2 days of age. HKx31 is an artificially reassortant influenza virus strain that shares all internal components, including NP and PA, with PR8 but differs in the exterior components hemagglutinin and neuraminidase—HKx31 expresses H3N2 and PR8 expresses H1N1 [39]. Therefore, only T cell memory to the internal components of either virus will be protective when mice are challenged with the other virus. Mice were infected with a LD_10_ dose of HKx31 or PR8 and then re-challenged 2 months later with a lethal dose of PR8 or HKx31, respectively. Mice first infected as pups demonstrated more weight loss than mice first infected as adults, although both groups were better protected than naïve animals, which were all euthanized by day 7 of infection upon reaching 20% weight loss (Figure 3A). Mice first infected as pups demonstrated a lower proportion of total CD8^+^ T cells in the BAL on day 7 post-infection (Figure 3B) as well as a lower proportion of NP (366–374)-specific cells among those CD8^+^ T cells at the same time point (Figure 3D). At day 7 post-infection, mice first infected as adults had a significantly higher proportion of activated CD8^+^ cells in the BAL fluid than mice first infected as pups (Figure 3C). However, over 80% of the NP^+^CD8^+^ T cells in the BAL fluid were activated, regardless of whether the mice were first infected as adults or as neonates (Figure 3E). Mice first infected as pups also had significantly fewer IFNγ-positive CD4^+^ and CD8^+^ T cells than mice first infected as adults (Figure 3F).

The secondary response immunodominance hierarchy in adult C57BL/6 mice has been shown to strongly favor NP (366–374) over PA (224–233) [30], and our data correspond well with this. Mice first infected as adults demonstrated significantly high proportions of NP (366–374)-specific CD8^+^ cells in the whole lung digest that arose around day 5 of infection and persisted through day 10 of infection (Figure 3G). In contrast, levels of PA (224–233)-specific cells remained easily detectable throughout the infection, but never rose significantly above starting proportions (Figure 3H). Mice first infected as pups also demonstrated clear proportions of NP-specific CD8^+^ T cells at days 7 and 10 post-infection (Figure 3G). Unlike the mice first infected as adults, mice first infected as pups developed appreciable proportions of PA (224–233)-specific CD8^+^ T cells by days 7 and 10 post-infection (Figure 3H). While both groups developed significant proportions of NP (366–374)- or PA (224–233)-specific CD8^+^ T cells by day 7 of infection, some of the mice first infected as adults showed expanded proportions of NP-specific cells as early as day 5 of infection (Figure 3G). These data suggest that primary influenza virus infection as a neonate alters the specificity and onset of antigen-specific cell expansion during influenza virus secondary challenges compared to secondary influenza virus infection in adults.

### 3.4. Adoptive Transfer of Bone-Marrow-Derived Dendritic Cells Alters CD8^+^ T Cell Immunodominance during Influenza Virus Infection and Improves Influenza Virus Clearance in Pups

**Figure 4:**
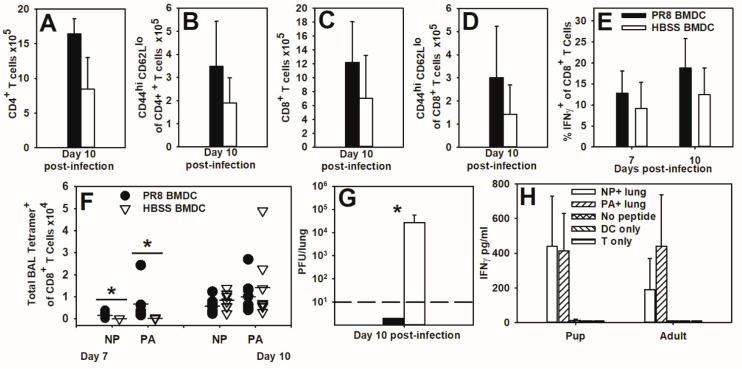
Adoptive transfer of PR8-infected BMDCs improves viral clearance and alters immunodominance during neonatal influenza virus infection. BMDCs were generated from C57BL/6 adult female mice and treated with LPS before incubation with the PR8 strain of the influenza virus or HBSS. Two-day-old C57BL/6 mice were injected i.p. with 10^4^ BMDCs before being infected i.n. with an LD_10_ dose of the PR8 strain of the influenza virus. The total lung CD4^+^ (**A**), total activated (CD44^+^ CD62L^lo^) CD4^+^ cells (**B**), total CD8^+^ (**C**), and total activated (CD44^+^ CD62L^lo^) CD8^+^ cells (**D**) were assessed by flow cytometry on day 10 post-infection. The proportion of CD8^+^ cells that were also IFNγ^+^ was assessed by flow cytometry on days 7 and 10 post-infection (**E**). Total NP or PA tetramer^+^ cells in the BAL were assessed by flow cytometry on days 7 and 10 post-infection (**F**). The viral burden at day 10 post-infection was assessed by plaque assay (**G**). Adult mice were infected with an LD_10_ dose of influenza and CD8^+^ cells were isolated from spleens on day 10 of infection. Lung digest from naïve 2 day-old or adult mice were co-cultured 1:5 with CD8^+^ cells and peptides. Supernatants were collected on day 3 and IFNγ concentration was assessed by ELISA (**H**). The data show the mean ± SD for two separate experiments with 4–8 mice per group. * *p* < 0.05.

Poor activation by neonatal dendritic cells has been implicated in many of the altered T cell responses observed in neonates [9,40,41]. To address whether neonatal dendritic cells are responsible for altered responses, BMDCs stimulated with LPS and incubated with the influenza virus or HBSS were transferred into 2-day-old pups before infection with an LD_10_ dose of the influenza virus. At day 10 post-infection, similar numbers of CD4^+^ or CD8^+^ T cells (Figure 4A,C), as well as activated (CD44^hi^ CD62L^lo^) T cells (Figure 4B,D) were found in the lung digests of mice that received either treatment. Both treatments were associated with similar proportions of IFNγ-producing CD8^+^ T cells in the lung digest at days 7 and 10 post-infection (Figure 4E), but PR8-infected BMDCs significantly increased NP- and PA-specific CD8^+^ T cells in the BAL of pups on day 7 post-infection. By day 10 post-infection, NP- and PA-specific CD8^+^ T cells are easily detectable in both treatment groups, but there were no significant differences (Figure 4F). Most intriguingly, pups that received PR8-infected BMDCs cleared the virus by day 10 of infection compared to control BMDCs (Figure 4G). These data suggest that providing activated adult-like dendritic cells to pups significantly increased the number of NP (366–374)-specific CD8^+^ T cells at day 7 post-infection and improved viral clearance in pups.

Using an in vitro assay, we determined that BMDCs present both NP (366–374) and PA (224–233) to adult CD8^+^ T cells (Figure S1). To assess whether adult NP-specific CD8^+^ T cells could be activated by pup lung cells, cells from the naïve pup or adult lung digest were co-cultured with CD8^+^ T cells isolated from the spleens of PR8-infected adult mice at day 10 post-infection and IFNγ production was measured at day 3 of culture by ELISA (Figure 4H). Pup and adult lung digest cells were able to activate adult CD8^+^ T cells through NP (366–374) or PA (224–233) peptide stimulation (Figure 4H). These data suggest that pups can present antigens to NP (366–374)-specific CD8^+^ cells.

### 3.5. Pups Vaccinated with Peptide-Loaded BMDCs Display Memory Responses to PA (224–233), but Not NP (366–374)

**Figure 5:**
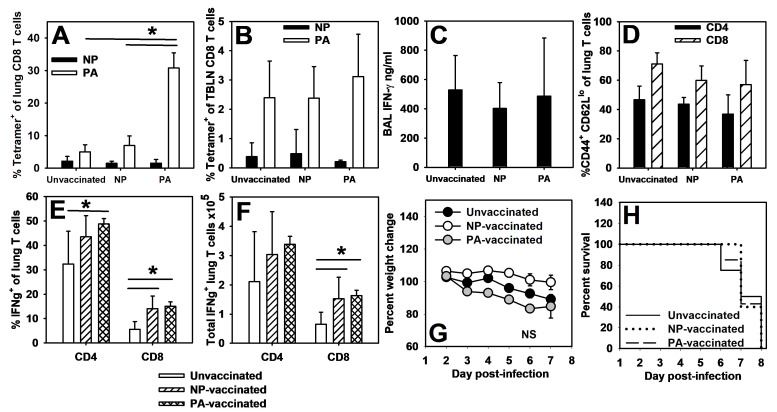
PA-vaccinated pups develop a PA-specific memory response, but are not protected from lethal infection. Two-day-old mice were injected i.p. with 10^4^ BMDCs pulsed with NP (366–374) or PA (224–233) and then infected 14 days later with an LD_10_ dose of the PR8 strain of the influenza virus. The percentage of CD8^+^ lymphocytes that are NP or PA tetramer^+^ in the lung digest (**A**) and TBLN (**B**) at day 7 post-infection was determined by flow cytometry. IFNγ was measured in the first wash of the BAL from day 7 post-infection by ELISA (**C**). The percentage of CD4^+^ and CD8^+^ lymphocytes that have an activated (CD44^hi^ CD62L^lo^) phenotype in the lung on day 7 post-infection was determined by flow cytometry (**D**). The proportion (**E**) and total numbers (**F**) of CD4^+^ and CD8^+^ lymphocytes that are IFNγ^+^ in the lung on day 7 post-infection were determined by flow cytometry. Two-day-old mice were also injected i.p. with 10^4^ BMDCs pulsed with NP (366–374) or PA (224–233) and then infected 14 days later with a lethal dose of the PR8 strain of the influenza virus. The pups were weighed daily (**G**) and survival was recorded (**H**). The data show the mean ± SD (**A**–**F**) or mean ± SEM (**G**) and represent two separate experiments with at least four mice per group. * *p* < 0.05.

Influenza virus-specific memory CD8^+^ T cell responses protect against subsequent infections resulting in reduced morbidity and mortality [42,43]. To test whether peptide vaccination in pups would protect against viral challenge, BMDCs generated from adult C57BL/6 bone marrow were incubated with either PA (224–233) or NP (366–374) before vaccinating 2-day-old pups via i.p. injection. Mice were infected with an LD_10_ dose of the PR8 influenza virus 14 days post-vaccination. Day 14 was chosen in order to evaluate mice early in life when their response to PR8 is dominated by PA-specific CD8 T cells. Antigen specificity, IFNγ production, and T cell activation were assessed in the whole lung digest, bronchoalveolar lavage (BAL), and the TBLN 7 days after infection. Pups vaccinated with PA (224–233) displayed elevated levels of PA-specific CD8^+^ T cells in the whole lung digest compared to unvaccinated or NP (366–374)-vaccinated pups, but NP (366–374)-vaccinated pups did not display elevated NP-specific CD8^+^ T cells compared to unvaccinated or PA (224–233)-vaccinated pups (Figure 5A). There were not significant differences between PA- or NP-specific CD8^+^ T cell populations in the TBLN (Figure 5B). The vaccinated and unvaccinated pups displayed similar IFNγ in the BAL regardless of the peptide used (Figure 5C). Additionally, vaccination did not impact the proportion of CD4^+^ or CD8^+^ T cells that were activated (CD44^+^ CD62L^lo^) (Figure 5D). The vaccinated animals had higher proportions (Figure 5E) and total numbers (Figure 5F) of IFNγ-producing CD8^+^ T cells than unvaccinated animals, and the PA-vaccinated pups had higher proportions of IFNγ-producing CD4^+^ cells than unvaccinated animals (Figure 5E).

These data suggest that PA vaccination results in a robust PA-specific response, but to ascertain whether this response offered protection during lethal challenge, NP- and PA-vaccinated mice as well as age-matched unvaccinated mice were infected 14 days post-vaccination with a lethal dose of influenza. All mice lost weight during infection (Figure 5G), and no mice survived the infection regardless of vaccination status (Figure 5H). These data suggest that although pups can respond to PA (224–233) peptide vaccination, this response is not protective during lethal influenza virus infection.

## 4. Discussion

CD8^+^ immunodominance during PR8 infections in adult C57Bl/6 mice is stable and predictable with NP and PA as dominant epitopes during primary infections [36]. Recent work, however, suggests that established immunodominance hierarchies may not hold in neonatal infections [32,33]. If neonates cannot be guaranteed to mirror adult hierarchies, then the patterns of neonatal immunodominance and the means to manipulate neonatal immunodominance must be understood independently of adult patterns. Subunit and peptide vaccines require predictable immunodominance hierarchies, and if infants do not respond to the same subunits as adults, then subunit T cell vaccines designed for adults could be ineffective in infant children.

We confirmed work by Carey et al. (2016) [32] that neonatal C57BL/6 mice did not respond to the adult dominant CD8^+^ T cell epitope of NP (366–374) during primary PR8 infections (Figure 1). Instead, pups responded to the PA (224–233) epitope (Figure 2). A secondary heterosubtypic infection in mice given primary infections as pups displayed altered CD8^+^ T cell response kinetics and immunodominance that correlated with increased morbidity (Figure 3). Transfer of adult BMDCs promoted the generation of NP (366–374)-specific CD8^+^ T cells in neonates, and transfer of adult influenza virus-infected BMDCs resulted in an earlier expansion of PR8-specific CD8^+^ T cells and improved viral clearance compared to transfer of uninfected adult BMDCs (Figure 4). Finally, pups vaccinated with PA (224–233) responded to the peptide, but the response offered no protection during influenza virus infection when vaccinated pups were infected two weeks later as weanlings (Figure 5).

Several studies of influenza virus and RSV infections have shown that neonatal CD8^+^ T cell immunodominance is shifted from adult hierarchies [32,33]. Tetramer analysis of pup whole lung digests and BALs demonstrated that pups produce PA (224–233)-specific CD8^+^ T cells but not NP (366–374)-specific CD8^+^ T cells during influenza virus infection (Figure 2B–E). PA-specific cells make up a similar proportion, and have similar total numbers of CD8^+^ T cells in pup and adult lungs, suggesting that this is the dominant antigen-specific population in neonatal anti-influenza virus responses (Figure 2F,H). Neonates typically display few lymphocytes in the alveolar spaces during influenza virus infection [11], but nearly 25% percent of CD8^+^ T cells in the BAL fluid were PA (224–233)-specific (Figure 2G), providing further evidence that PA-specific cells are a major component of the neonatal CD8^+^ T cell response. However, other studies have suggested that 7-day-old and 2-week-old pups can respond to NP (366–374), so the absence of these cells in our study may represent a phenomenon of very early life [32,44].

A study from Rius et al. (2018) suggests that MHC-peptide tetramers can underestimate true antigen-specific cell populations, especially for low affinity T cells such as tumor-infiltrating lymphocytes or autoreactive T cells [45]. It is possible that the lack of tetramer staining in neonatal lung digests and BALs is due to low affinity anti-NP cells instead of an absence of those cells. However, the NP-specific CD8^+^ T cells produced by pups that received BMDCs were detectable with tetramers, suggesting that the lack of NP tetramer staining in normal neonatal infections is due to a lack of NP-specific cells and not an inability to detect neonatal NP-specific cells (Figure 4F). Even without BMDCs, pups do develop detectable numbers of NP (366–374)-specific CD8^+^ T cells well after the virus is cleared (Figure 1G). Although these cells appear too late to participate in a primary response, they may impact secondary responses.

Skewed immunodominance in pups could shift the specificity of CD8^+^ T cells in secondary responses as well, leading to unpredictable protection during memory responses later in life. Mice first infected as adults show a strong NP (366–374)-dominant secondary response, as previously described [30,38]. Also consistent with previous work, mice first infected as pups displayed robust CD8^+^ T cell responses [32], and intriguingly, many displayed NP dominance. The pattern, however, was less predictable than in mice first infected as adults (Figure 3G). A few of the animals first infected as pups were even PA (224–233)-dominant in the secondary response, maintaining the neonatal immunodominance pattern (Figure 3H). Additionally, although both groups exposed to a heterosubtypic infection displayed similar levels of total NP (366–374)- and PA (224–233)-specific CD8^+^ T cells by day 7 of infection, there was an earlier onset of expansion in many of the mice first infected as adults (Figure 3G). The timing of this shift in expansion had some variability, but three of the four trials performed showed earlier onset of antigen-specific CD8^+^ T cell populations in the mice first infected as adults. These data suggest that primary infection as a neonate will promote a slower antigen-specific secondary CD8^+^ response than primary infection as an adult, while still providing some protection during secondary infections (Figure 3G,H).

Given that neonates seemed capable of responding to NP (366–374) in a few individual animals, we wanted to find a way to force a more consistent NP (366–374)-specific response in pups. Neonatal dendritic cells have been shown to be much less stimulatory than adult dendritic cells in vivo due to a number of factors, including the lower expression of costimulatory molecules and MHC class I and II [9]. We reasoned that during influenza virus infections, BMDCs might provide sufficient stimulation to promote a more adult-like CD8^+^ T cell response. Pups that received LPS-stimulated BMDCs developed an NP (366–374)-specific population whether or not the dendritic cells were loaded with PR8 (Figure 4F), supporting the idea that neonatal dendritic cells may be responsible for the pups’ altered hierarchies [40,41]. It is not surprising that both unloaded and PR8-loaded BMDCs could promote NP-specific CD8^+^ T cell expansion, as unloaded BMDCs will also take up PR8 during an infection. However, while both BMDC treatments promoted NP-specific CD8^+^ populations at the peak of infection, PR8-loaded BMDCs did significantly improve the time needed to clear the virus (Figure 4G). This correlates with an increase in total NP- and PA-specific CD8^+^ T cells at day 7 post-infection in the BAL (Figure 4F). Given that there were no differences in the total CD4^+^ or CD8^+^ T cells, activation status of those cells, or IFNγ production, we suspect that this altered kinetics of antigen-specific CD8^+^ T cell populations may be an important factor in explaining the improved viral clearance that PR8-loaded BMDCs provided (Figure 4A–E). Finally, co-cultured pup lung tissue is able to activate memory NP-specific CD8^+^ T cells taken from PR8-infected adult mice, suggesting that antigen-specific T cells can be reactivated successfully by neonatal lung cells (Figure 4H). It is important to note that a defect in antigen processing cannot be ruled out, as pup lung tissue was presenting NP (366–374) rather than processing the whole NP protein and presenting the appropriate peptide.

Altered immunodominance in primary infections has important implications for vaccination efficacy. Subunit vaccines rely on immunodominance to predict likely antigens that will provoke effective B or T cell responses across a population. The success of this strategy relies on two things: first, immune cells must be able to respond to a subunit antigen, and secondly, the response to that antigen must be protective. Unfortunately, the altered immunodominance in neonates could compromise responses to subunit vaccines designed for adults. A potential solution for shifted immunodominance is simply to target age-appropriate antigens for vaccines used in neonatal populations, but studies on herpes simplex virus 1 (HSV-1) have suggested that neonatal infections can “lock in” suboptimal immunodominance hierarchies for subsequent adult infections [46]. Additionally, previous work has also shown that PA-specific CD8^+^ T cells are not effective in protecting adult mice from influenza virus challenge [30,38]. These studies demonstrated that although NP- and PA- specific responses can be generated by dendritic cells presenting each peptide, influenza-virus-infected respiratory epithelium only presents NP (366–374) and not PA (224–233). We wanted to determine not only whether pups could respond to a peptide vaccine, but also whether a response would offer protection during lethal challenge.

Pups responded only to PA (224–233) when vaccinated with NP (366–374)- or PA (224–233)-peptide-pulsed BMDCs (Figure S2A). The PA (224–233)-specific response exhibited by neonates expanded appropriately during subsequent challenge, suggesting that the peptide vaccine succeeded in establishing PA-specific CD8^+^ T cell memory in pups (Figure 5A). NP vaccination of pups did not induce a similar expansion of NP-specific cells (Figure 5A,B). Neither vaccine increased T cell activation (Figure 5D), but both vaccinated groups had more IFNγ-producing cells than the unvaccinated animals (Figure 5E,F). This was not different between NP and PA vaccination, suggesting that this increase is not related to the expansion of PA-specific cells in PA-vaccinated pups. This is consistent with other work showing that pups respond in a more Th1 manner when given adult dendritic cells [16,17]. Despite demonstrating a robust PA-specific CD8^+^ T cell response, PA (224–233)-vaccinated pups were not protected from weight loss or mortality during lethal influenza infection (Figure 5G,H).

In conclusion, these data show a consistent shift in neonatal C57BL/6 CD8^+^ T cell immunodominance during PR8 infection that influences, but does not guarantee, the hierarchy of secondary infections. Additionally, this altered hierarchy can be manipulated by altering the quality of dendritic cell stimulation available to neonatal CD8^+^ T cells, such as that provided by BMDCs. Most importantly, PA (224–233) vaccination does not protect pups during influenza infection. These data suggest that other influenza virus epitopes may be critical in bolstering the immune response to influenza virus. Likewise, age should be considered when designing subunit vaccines for infants, and with mechanisms to ensure proper costimulation, it may be possible to guarantee a protective immune response to the same targets in both neonates and adults. Manipulating neonatal dendritic cells is a promising path to accomplish this goal.

## Data Availability

The data presented in this study are contained in this article. Further inquiries can be directed to the corresponding author.

## Supplementary Materials

The following supporting information can be downloaded at: https://www.mdpi.com/article/10.3390/v16081271/s1, Figure S1: Bone marrow derived dendritic cells present NP(366–374) and PA(224–233) to CD8^+^ T cells; Figure S2: Pups respond to PA(224–233)-loaded BMDCs, but not NP(366–374)-loaded BMDCs.
