# Supplementary material for: The Altered Neonatal CD8+ T Cell Immunodominance Hierarchy during Influenza Virus Infection Impacts Peptide Vaccination
Source: Viruses. 2024 Aug 9;16(8):1271. doi: 10.3390/v16081271 (PMC11359775; doi:10.3390/v16081271)
Supplement: Supplementary file 1 [file viruses-16-01271-s001.zip › viruses-3129805-supplementary.pdf]

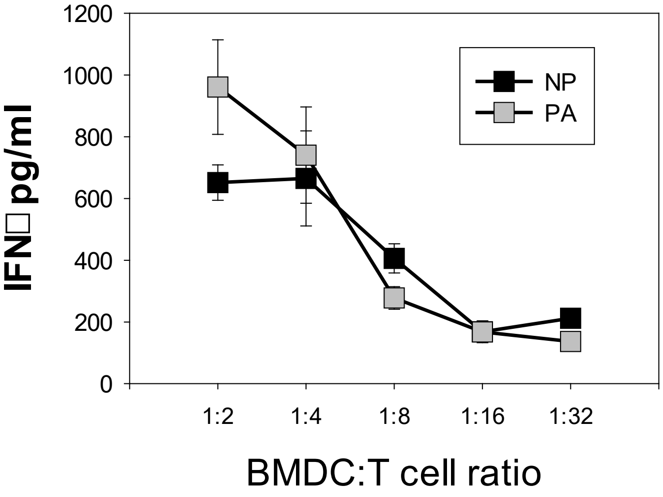

Supplementary Figure S1. Bone marrow derived dendritic cells present NP (366-374) and PA (224-233) to CD8$^+$ T cells.

Adult mice were infected with an LD_{10} dose of influenza virus and CD8$^+$ T cells were isolated from spleens on day 10 of infection. Adult CD8$^+$ T cells were co-cultured with LPS stimulated BMDCs and NP (366-374) or PA (224-233) peptide and supernatants were collected on day 3 of culture. IFNγ concentration was measured by ELISA. Data represent mean ± SEM for 3 replicate wells and 2 separate experiments.

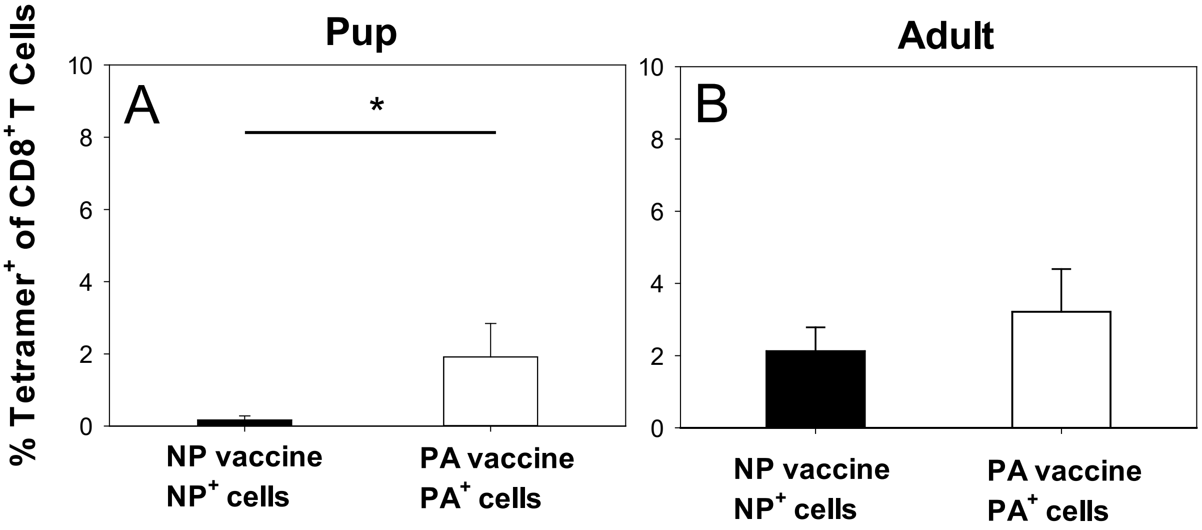

Supplementary Figure S2. Pups respond to PA (224-233) loaded BMDCs, but not NP (366-374) loaded BMDCs.
Two day-old and adult mice were injected i.p. with 10^4 or $5x10^4$ BMDCs pulsed with either NP (366-374) or PA (224-233). Spleens were obtained 14 days after vaccination and pup (A) and adult (B) $CD8^+$ T cells were assessed for antigen specificity by flow cytometry. Data represent mean ±SD for 2 separate experiments with at least 3 mice per group. * $p<0.05$.
